# Injuries to the Peripheral Nerves as Observed in Soldiers Returned to This Country

**Published:** 1919

**Authors:** 


***Injuries to the Peripheral Nerves as Observed in Soldiers Returned to this Country.*** By ALEXANDER GIBSON. Address before the North Dakota State Medical Association. *The Lancet,* October 15, 1918.

In military work, injuries to the peripheral nerves are "astonishingly numerous " either as pathological entities standing alone, or more frequently as concomitants to injuries of the bone or soft parts. In these injuries there are a number of complicating factors, of which the most important are the presence of scar-tissue, and the muscular complications.

In any case showing paralysis or loss of sensation, the following points must be considered.

1. Is there a real nerve lesion?
2. If so, where is it?
3. Is it complete or incomplete?
4. If it is complete, is it recovering, or is there no progress toward recovery?

In considering the history of the cases, the following points are of importance.

1. Date of wound.
2. Date of complete healing.
3. Previous treatment.

The examination of the patient must include:

*First.* General inspection; note the patient's attitude; watch him in action; look for alterations of shape, color, contour.

*Second.* Examine the sensory disturbance. Test with cotton wool for epicritic sensation; with a pin for appreciation of sharp and blunt; with the end of a fountain-pen for deep sensation. Map out carefully the areas of loss.

*Third.* Investigation of motor disturbances. The examiner must know the muscles supplied by each nerve and attempt to make each muscle act independently. Some experience is necessary.

*Fourth.* Electrical investigation. Muscles must be tested for their response to faradism and galvanism. If there is response to galvanism, the muscle fibers are still excitable. If no response, the process of degeneration is pronounced. If careful examination shows that the lesion is complete, a restoration of function by surgical means is indicated.

In the incomplete lesion, it is often difficult to determine the possibility and progress of recovery, and to decide whether the treatment should be operative or non-operative. We can ascertain the fact of regeneration or otherwise by tapping the nerve sharply at some point below the lesion; if regenerating fibers are present there will be a tingling sensation passing to the peripheral distribution of the nerve.

The rate of progress in regeneration can be determined by making careful surveys at definite intervals while the patient is under treatment.

In the cases of complete interruption of the nerve, the chief symptoms are :

1. Sensory changes : anesthesia in the cutaneous distribution of the nerve; deep anesthesia usually widespread; muscles not sensitive to pressure. Trophic signs may be present.

2. Motor changes : complete loss of tone and absolute paralysis of every muscle supplied by the nerve below the lesion.

3. Electrical : excitation of the nerve above the lesion produces no response. For a short time after section, excitation below the lesion will produce a response; the response of the muscle to galvanism lasts longer than the nerve response to faradism.

In addition to these symptoms, there are often some of the phenomena of irritation.

The cases of incomplete interruption show more varied symptoms which are more difficult to interpret correctly. There are symptoms of irritation of some of the fibers, symptoms of compression, and frequently symptoms of irritation. A common condition is the presence of a tender scar directly continuous with a nerve trunk.

If non-operative treatment proves successful, the signs of recovery looked for are :

1. Sensory recovery : deep pressure will practically always produce a response before superficial stimulation. The area of muscle is usually hypersensitive.

2. The return of deep sensation is practically always accompanied by the filling out of atrophied spaces.

3. From a very early period the presence of sensitiveness to percussion at a level below the lesion can be made out.

4. Motor recovery is usually somewhat later. In the non-operative treatment, the most important principle is that the muscles must be maintained as long as possible in good condition. This involves both active and passive treatment. Very light massage and mild galvanism should be applied daily for months, if necessary. On the other hand the paralyzed muscle should never be overstretched. The prevention of overstretching applies without exception to every peripheral nerve injury, and usually means the use of a different splint for each type of case.

In the surgical treatment various types of operation are called for. These include :

1. Neurolysis. Simple liberation from scar tissue.

2. Partial suture. This is the operation of choice when possible.

3. End to end suture. This is the operation that will have to be performed most frequently. The author uses plain catgut for these sutures. Tension of the nerve should be avoided; for this purpose the flexion of adjoining joints is often indicated.

Where coaptation is not possible, various devices have been tried, among which the author mentions :

1. Lateral implantation of proximal and distal nerves into an intact nerve.

2. Nerve grafts : using a portion of a sensory nerve to act as a bridge.

3. The use of a catgut bridge.

Where nerve function cannot be restored tendon transplantation may be performed.

The prognosis of peripheral nerve injuries is not so gloomy as has been supposed. A large number, probably 60 to 70 $^0/_0$, attain a large measure of recovery without operation; these are all cases of incomplete division. Of those operated, the great majority, probably 90 o/o, will show improvement, which may proceed to complete recovery if adequate non-operative treatment is given.

